# Development and validation of a disulfidptosis-related scoring system to predict clinical outcome and immunotherapy response in acute myeloid leukemia by integrated analysis of single-cell and bulk RNA-sequencing

**DOI:** 10.3389/fphar.2023.1272701

**Published:** 2023-11-20

**Authors:** Fangmin Zhong, Junyao Jiang, Fang-Yi Yao, Jing Liu, Xu Shuai, Xin-Lu Wang, Bo Huang, Xiaozhong Wang

**Affiliations:** Department of Clinical Laboratory, Second Affiliated Hospital of Nanchang University, Nanchang, China

**Keywords:** disulfidptosis, acute myeloid leukemia, tumor microenvironment, prognosis, immunotherapy

## Abstract

**Background:** Disulfidptosis is a metabolically relevant mode of cell death, and its relationship with acute myeloid leukemia (AML) has not been clarified. In this study, disulfidptosis scores were computed to examine the potential biological mechanisms.

**Methods:** Consensus clustering was applied to detect disulfidptosis-related molecular subtypes. The least absolute shrinkage and selection operator (LASSO) regression analysis was used to construct a DRG prognostic model.

**Results:** DRGs are upregulated in AML and associated with poor prognosis. The higher the disulfidptosis activity score, the worse the clinical outcome for patients, accompanied by increased immune checkpoint expression and tumor marker pathway activity. The two molecular subtypes exhibited distinct prognoses and tumor microenvironment (TME) profiles. A prognostic risk score model was established using six DRGs, and the AML cohort was divided into high- and low-risk score groups. Patients in the high-risk group experienced significantly worse prognosis, which was validated in seven AML cohorts. Receiver Operating Characteristic (ROC) curve analysis indicated that the area under the curve values for risk score prediction of 1-, 3-, and 5-year survival were 0.779, 0.714, and 0.778, respectively. The nomogram, in conjunction with clinicopathological factors, further improved the accuracy of prognosis prediction. The high-risk score group exhibited a higher somatic mutation frequency, increased immune-related signaling pathway activity, and greater immune checkpoint expression, suggesting a certain degree of immunosuppression. Patients with advanced age and higher cytogenetic risk also had elevated risk scores. According to drug prediction and AML anti-PD-1 therapy cohort analysis, the low-risk score group displayed greater sensitivity to chemotherapy drugs like cytarabine and midostaurin, while the high-risk score group was more responsive to anti-PD-1 therapy. Finally, clinical samples were collected for sequencing analysis, confirming that the progression of myeloid leukemia was associated with a higher risk score and a negative disulfidptosis score, suggesting that the poor prognosis of AML may be associated with disulfidptosis resistance.

**Conclusion:** In conclusion, a systematic analysis of DRGs can help to identify potential disulfidptosis-related mechanisms and provide effective new biomarkers for prognosis prediction, TME assessment, and the establishment of personalized treatment plans in AML.

## Introduction

Acute myeloid leukemia (AML) is a hematologic tumor that originates from hematopoietic stem cells (HSCs) (Shimony et al., 2023). The prognosis for patients with AML is extremely poor. Currently, the conventional treatment involves induction chemotherapy. However, due to variations in age, individual physical condition, and disease heterogeneity, this treatment often yields suboptimal responses, frequently resulting in relapse or drug resistance (Bhansali et al., 2023). Therefore, finding new therapeutic targets and prognostic markers is imperative for the management of patients with AML.

Regulatory cell death (RCD) refers to a mode of cell death that is governed by specific molecular pathways and can be regulated through artificial means, such as genetics or pharmacology (Galluzzi et al., 2018). The controlled occurrence of RCD plays a pivotal role in bodily development and cellular homeostasis. Conversely, dysregulated RCD is closely associated with various diseases, including cancer. Evading cell death is recognized as a fundamental hallmark of cancer. The presence of apoptosis resistance in tumor cells has prompted researchers to investigate alternative RCD mechanisms (Mohammad et al., 2015). Non-apoptotic RCD encompasses autophagy, ferroptosis, pyroptosis, and necroptosis. Among these, ferroptosis is a form of RCD induced by iron-dependent lipid peroxidation discovered in recent years, which has a unique morphology and mechanism of occurrence (Dixon et al., 2012). Recent studies have shown that certain cancer cells that are resistant to conventional therapies are particularly susceptible to ferroptosis (Zhang et al., 2022). Ferroptosis regulated by solute carrier family 7 member 11 (SLC7A11; also known as xCT)-mediated cystine uptake plays a key role in promoting glutathione biosynthesis and mitigating oxidative stress (Koppula et al., 2021). However, a 2017 study demonstrated that SLC7A11 significantly promotes cell death under glucose-starvation conditions (Goji et al., 2017; Koppula et al., 2017; Shin et al., 2017), contradicting prior research findings. In 2020, Liu et al. (2020) uncovered the mechanism by which SLC7A11-mediated reduction of ingested cystine to cysteine depends heavily on reduced nicotinamide adenine dinucleotide phosphate (NADPH) generated by the glucose-pentose phosphate pathway. Consequently, in glucose -starvation conditions, NADPH is depleted in cells overexpressing SLC7A11, leading to abnormal accumulation of disulfide stress, such as cystine, which triggers rapid cell death. Recently, Gan et al. revealed the mechanism behind disulfide stress-induced cell death and coined this novel mode of cell demise as disulfidptosis (Liu et al., 2023).

In the context of AML, inhibiting SLC7A11 can enhance the effects of chemotherapy by preventing cystine uptake (Pardieu et al., 2022). Furthermore, AML cell growth and proliferation also depend on more active glucose metabolism (Chen et al., 2014). Therefore, inducing disulfidptosis through glucose starvation as a treatment strategy for AML holds potential therapeutic value. In this study, we conducted a comprehensive analysis of the expression patterns of disulfidptosis-related genes (DRGs) in AML samples. We computed disulfidptosis-related scores using single-sample gene set enrichment analysis (ssGSEA) and analyzed the relationship between DRGs and AML prognosis, pathway activity, and the tumor microenvironment (TME). Additionally, we developed a risk score model to predict the prognosis and immunotherapy response of patients with AML. This research furnishes a more substantial theoretical foundation and data support for the exploration of AML disulfidptosis and provides personalized guidance for the clinical treatment and prognosis evaluation of AML.

## Materials and methods

### Data acquisition and processing

A total of 1,653 AML samples and 337 normal samples were included in this study. AML samples included The Cancer Genome Atlas-Acute Myeloid Leukemia (TCGA-LAML) cohort and seven GEO cohorts (GSE10358-GPL570, GSE12417-GPL96, GSE12417-GPL570, GSE37642-GPL96, GSE37642-GPL570, GSE71014-GPL10558), GSE14468-GPL570) (Supplementary Table S1), and normal samples were Genomic tissue expression (GTEx)-whole blood cohort. For GEO cohort data from the affymetrix platform, raw “CEL” files were downloaded and normalized with the use of the robust multiarray averaging (RMA) method, whereas microarray data from the other platforms were directly downloaded with a normalized matrix file. TCGA-LAML and GTEx RNA-seq data (RSEM TPM) were downloaded from the UCSC XENA database (https://xenabrowser.net/datapages/). The “human.gtf” file was adopted to raw matrix annotation. All data were analyzed using R x64 and the associated R Bioconductor software package, and the data information is shown in Supplementary Table S1. Ten DRGs were retrieved from the study by Gan et al., of which, SLC7A11, SLC3A2, RPN1, and NCKAP1 are drivers, and GYS1, NDUFS1, OXSM, LRPPRC, NDUFA11, and NUBPL are suppressors.

### Calculation of disulfidptosis-related scores

We used the ssGSEA algorithm to calculate enrichment scores for disulfidptosis drivers and suppressors, defined as disulfidptosis positive score and negative score, respectively, and subtracted the negative score from the positive score to obtain disulfidptosis activity score (Subramanian et al., 2005).

### Consensus cluster analysis of DRGs

Based on the expression profiles of 10 DRGs, the “ConsensusCluster” package was used to perform unsupervised clustering of the TCGA-LAML dataset by consensus clustering method (Wilkerson and Hayes, 2010), and two cluster subtypes with significant differences were obtained. We performed 1,000 replicates to ensure stable and reliable clustering.

### Weighted correlation network analysis (WGCNA)

WGCNA can assess patterns of gene expression correlations and perform methods for visualization of co-expression networks. We used the “WGCNA” software package to identify genes associated with disulfidptosis scores in the TCGA-LAML cohort (Langfelder and Horvath, 2008). Pearson correlation analysis was used for adjacency matrix formation for all paired genes, and a scale-free topology of the adjacency matrix was implemented based on the optimal soft threshold power. Then, we further transform the adjacency matrix into the topological overlap matrix (TOM). Based on the TOM difference measure, a minimum module size of 30 and a cut height of 0.2 were set to partition genes with similar expression patterns into the same modules by average linkage hierarchical clustering. Then, the correlation between module eigengenes (MEs) and disulfidptosis score was evaluated, and the modules that met the study purpose were determined according to the degree of correlation.

### Pathway enrichment analysis

For the target module genes identified by WGCNA, Kyoto Encyclopedia of Genes and Genomes (KEGG) enrichment analysis was used to identify gene functions. The Gene Set Variation Analysis (GSVA) algorithm was used to calculate the activity scores of individual gene sets in different samples, and the Gene Set Enrichment Analysis (GSEA) algorithm was used to evaluate the difference in pathway activity between patients with high- and low-risk score groups to analyze the biological differences among patients with different risk scores. We used q value < 0.05 as a threshold for significant enrichment.

### Assessment of TME and immune cell infiltration

We used the ESTIMATE algorithm (Yoshihara et al., 2013) to evaluate the immune and stromal scores for each AML sample and applied the CIBERSORT algorithm (Newman et al., 2015) to determine the proportion of immune cell subsets in each sample.

### Construction of risk score model

We performed univariate Cox regression analysis of disulfidptosis activity score related genes based on *p* < 0.01 was used to identify the DASRGs significantly related to AML prognosis. In order to limit the influence of multicollinearity between variables, the least absolute shrinkage and selection operator (LASSO) regression analysis was used to further reduce the dimension and screen out the optimal variables to prevent instability caused by model estimation distortion, so as to construct an accurate prognostic risk score model. The risk score for each sample was obtained by multiplying the expression value of each model gene with its corresponding coefficient and adding it. Then, the risk scores of all patients were ranked, and AML patients were divided into high-risk score group and low-risk score group based on the optimal cut-off value, and the differences in clinicopathological factors and biological characteristics between the two groups were further analyzed.

### Assessment of mutation and treatment sensitivity

We download the somatic mutation data from the TCGA database (https://portal.gdc.cancer.gov/), and compared the mutation differences in high- and low-risk score groups. The “pRRophetic” package was used to predict the half maximal inhibitory concentration (IC50) of AML samples to commonly used therapeutic drugs (Geeleher et al., 2014). A smaller IC50 value indicates a better treatment effect. We further used the SubMap (https://cloud.genepattern.org/gp) algorithm to predict the response of different risk score groups to anti-PD-1 and anti-CTLA4 immune checkpoint inhibitors.

### Single-cell RNA-seq data processing

We downloaded AML single-cell sequencing data containing 21 cell types (GSE116256) from the GEO database, as well as another group of AML Single-cell sequencing data in the context of PD-1 blocking (GSE198052). We referred to previously published literature related to single cells (Jiang et al., 2022; Zheng et al., 2023). The 10 × scRNA-seq data were processed by R software according to a standardized procedure. The original gene expression matrix was introduced into the “Seurat” package for processing, only genes expressed in at least three single cells, and cells with unique molecular identifiers (UMI) counts <200 were removed. Moreover, only cells expressing more than 1,500 genes and less than 6,000 genes were included. The percentage of mitochondrial or ribosomal genes was calculated for each cell, and cells with more than 20% mitochondrial gene expression were considered low quality cells and also not subjected to downstream analysis. Then, normalized counts were obtained by using the library size normalization of the original matrix, and the top 2,000 genes with a large coefficient of variation were obtained by using the “FindVariableFeatures” function. After z-score processing, principal component analysis (PCA) was performed based on high-variable genes. The uniform manifold approximation and projection (UMAP) algorithm was used to realize the visualization of clustering. Cell types were referred to the annotation file provided by van Galen et al. (2019).

### Myeloid leukemia clinical sample collection

Clinical samples were collected in accordance with the Declaration of Helsinki and institutional guidelines, and informed consent was obtained from each patient and healthy volunteer. Ethical approval was obtained from the Ethics Committee of the Second Affiliated Hospital of Nanchang University [No. review. (2018) No. (092)], and all experimental protocols and methods were performed in accordance with relevant protocols and regulations. Five samples from patients with newly diagnosed chronic myeloid leukemia without any previous treatment, five samples from patients in blast crisis, and five normal samples from healthy volunteers were collected according to the World Health Organization classification of tumors of hematopoietic and lymphoid tissues. Detailed details of sample collection, next-generation sequencing, and processing procedures are available in our previous publications (Li et al., 2020).

### Statistical analysis

Wilcoxon and Kruskal–Wallis tests were used for between-two and multiple-group comparisons, respectively. The “survminer” package was used to determine the optimal cut-off value. The number of patients in a single risk group was set to be no less than 30% of the total population. Kaplan-Meier survival analysis was performed using the log-rank test. The receiver operating characteristic (ROC) curve was used to evaluate the specificity and sensitivity of the risk score, and the area under the curve (AUC) was determined. Bilateral *p* < 0.05 was considered statistically significant.

## Results

### Analysis of DRG expression and scoring patterns in bulk RNA-sequencing

**FIGURE 1:**
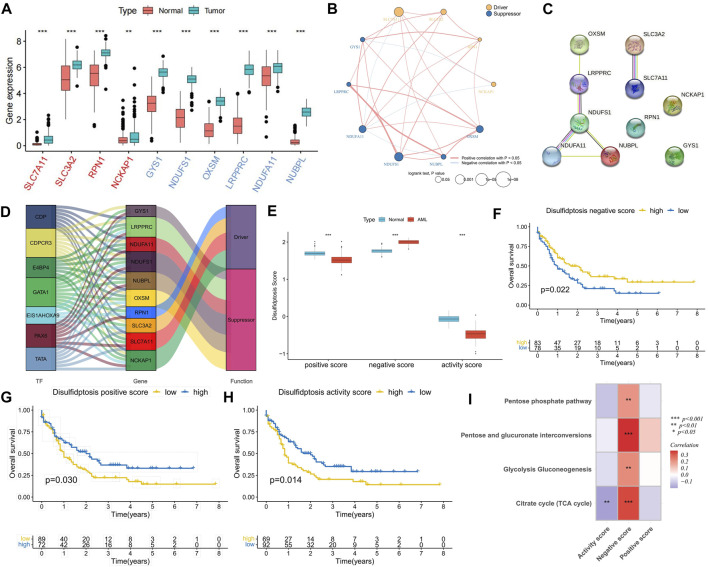
Expression characteristics of disulfidptosis-related genes (DRGs) and correlation analysis of disulfidptosis score. **(A)** Differential expression analysis of DRGs between AML samples and normal samples; The red ones are drivers and the blue ones are suppressors. **(B)** Correlation analysis of DRGs expression and its relationship with AML prognosis. **(C)** PPI network connectivity diagram of DRGs. **(D)** Potential regulatory relationships between transcription factors (TFs) and DRGs. **(E)** Differences in disulfidptosis score between AML samples and normal samples. **(F–H)** K-M curve analysis of positive, negative, and activity scores for disulfidptosis. Log-rank test. **(I)** Correlation analysis between disulfidptosis score and glucose metabolic pathway activity. (*p < 0.05; **p < 0.01; ***p < 0.001).

In comparison with normal samples, DRGs exhibited upregulated expression in AML samples, suggesting potential crosstalk between DRGs and AML (Figure 1A). Expression correlation analysis indicated positive correlations among most DRGs, with suppressor *NDUFA11* showing a negative correlation with drivers *SLC7A11* and *NCKAP1*. Additionally, *NCKAP1* and *GYS1* exhibited a negative correlation (Figure 1B), suggesting the presence of an antagonistic regulatory mechanism in disulfidptosis. Cox regression analysis identified DRGs as risk factors, except for *NUBPL* (Figure 1C). K-M curve analysis demonstrated that high expression groups of *SLC7A11*, *SLC3A2*, *OXSM*, *NDUFA11*, and *NDUFS1* had significantly worse prognoses than low expression groups, while the high expression group of *NUBPL* had a significantly better prognosis (Supplementary Figures S1A, B). PPI network analysis revealed *NDUFS1* as the hub gene among the suppressors (Figure 1C). Furthermore, a set of TFs with potential regulatory roles with DRGs was identified (Figure 1D). Using the GSVA algorithm, disulfidptosis positive, negative, and activity scores were computed. Compared to normal samples, AML samples exhibited higher disulfidptosis positive and activity scores and lower negative scores, indicating AML cell resistance to disulfidptosis (Figure 1E). K-M curve analysis showed that patients in both high positive and activity score groups had worse prognoses than those in low score groups, with the opposite observed for the negative score (Figures 1F–H). The occurrence of disulfidptosis was found to be influenced by glucose starvation, with glucose metabolic pathways such as citrate cycle (TCA cycle), glycolysis gluconeogenesis, pentose and glucuronate interposition, and pentose phosphate pathway enrichment scores being significantly positively correlated with disulfidptosis negative score. Conversely, the TCA cycle was significantly negatively correlated with the positive score (Figure 1I), implying that increased glucose metabolism activity is unfavorable for disulfidptosis occurrence.

### Validation of DRG expression and scoring patterns in single-cell sequencing

**FIGURE 2:**
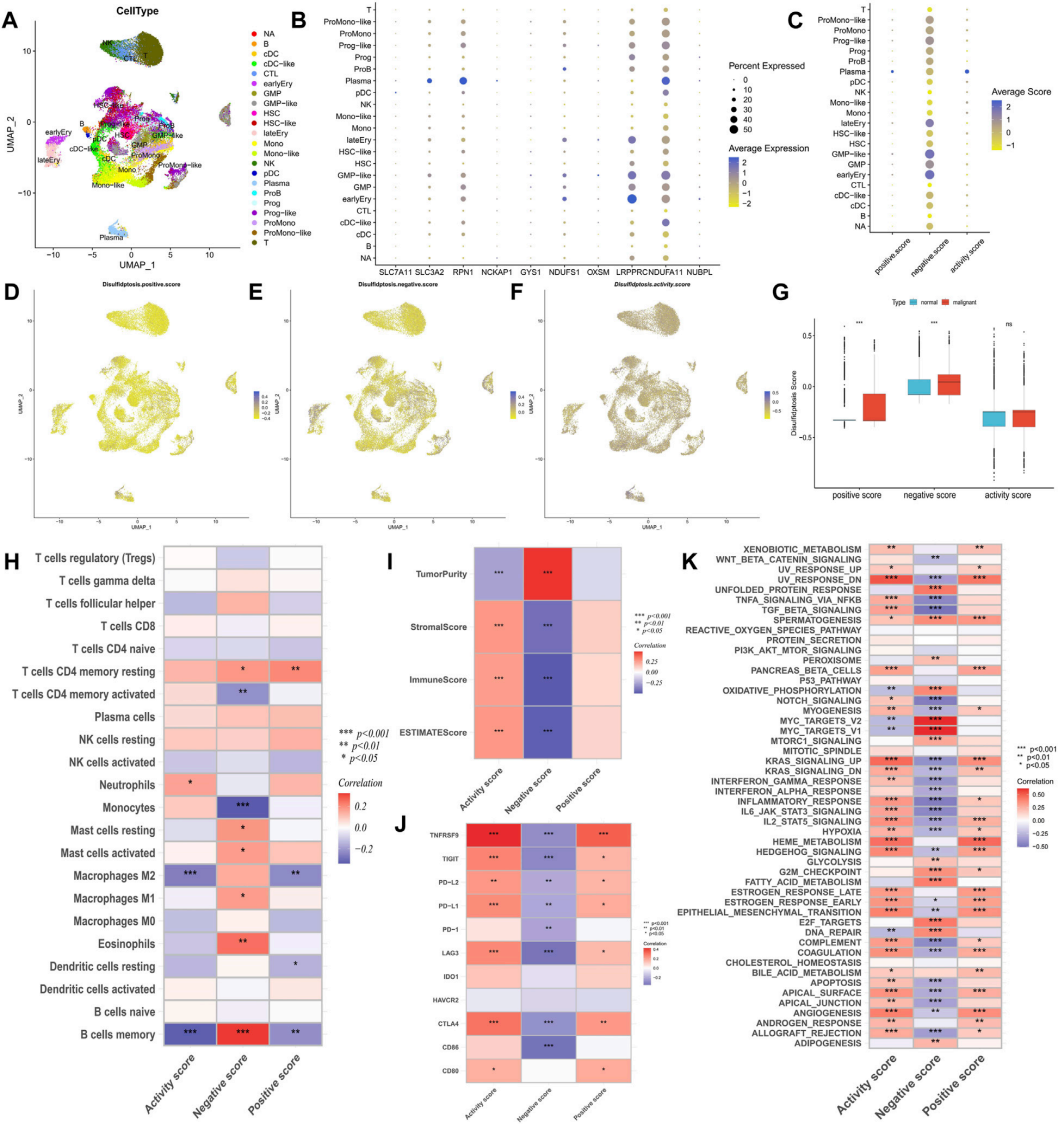
Analysis of potential biological mechanisms of disulfidptosis. **(A)** UMAP analysis of the AML single-cell sequencing dataset GSE116256 shows the distribution characteristics of all cell types. **(B,C)** The bubble maps show the expression characteristics of disulfidptosis genes and scores in all cell types. **(D–F)** Heatmaps showe the distribution characteristics of disulfidptosis scores in all cells. **(G)** Differences in disulfidptosis scores between normal cells and AML malignant cells. **(H–K)** Correlation analysis of disulfidptosis score with immune cell infiltration **(H)**, TME score **(I)**, immune checkpoint expression **(J)**, and tumor marker pathway activity **(K)**. (*p < 0.05; **p < 0.01; ***p < 0.001).

The GSE116256 cohort included bone marrow samples from 16 patients with AML and 5 healthy participants, encompassing 21 cell types (Figure 2A). Among these, six types of AML malignant cells were identified as HSC-like, progenitor-like, granulocyte-monocyte-progenitor (GMP)-like, promonocyte-like, monocyte-like, and conventional dendritic cell-like. Expression profile analysis revealed that disulfidptosis suppressor genes like *LRPPRC* and *NDUFA11* exhibited higher detectable expression rates in single cells, particularly in GMP-like cells (Figure 2B). Furthermore, the calculation of disulfidptosis scores in various cells showed that AML malignant cells, especially GMP-like cells, had higher negative scores (Figures 2C–F). Additionally, malignant cells demonstrated higher positive scores, resulting in no significant difference in activity scores between them and normal cells (Figure 2G).

### Exploration of the potential mechanism of disulfidptosis

**FIGURE 3:**
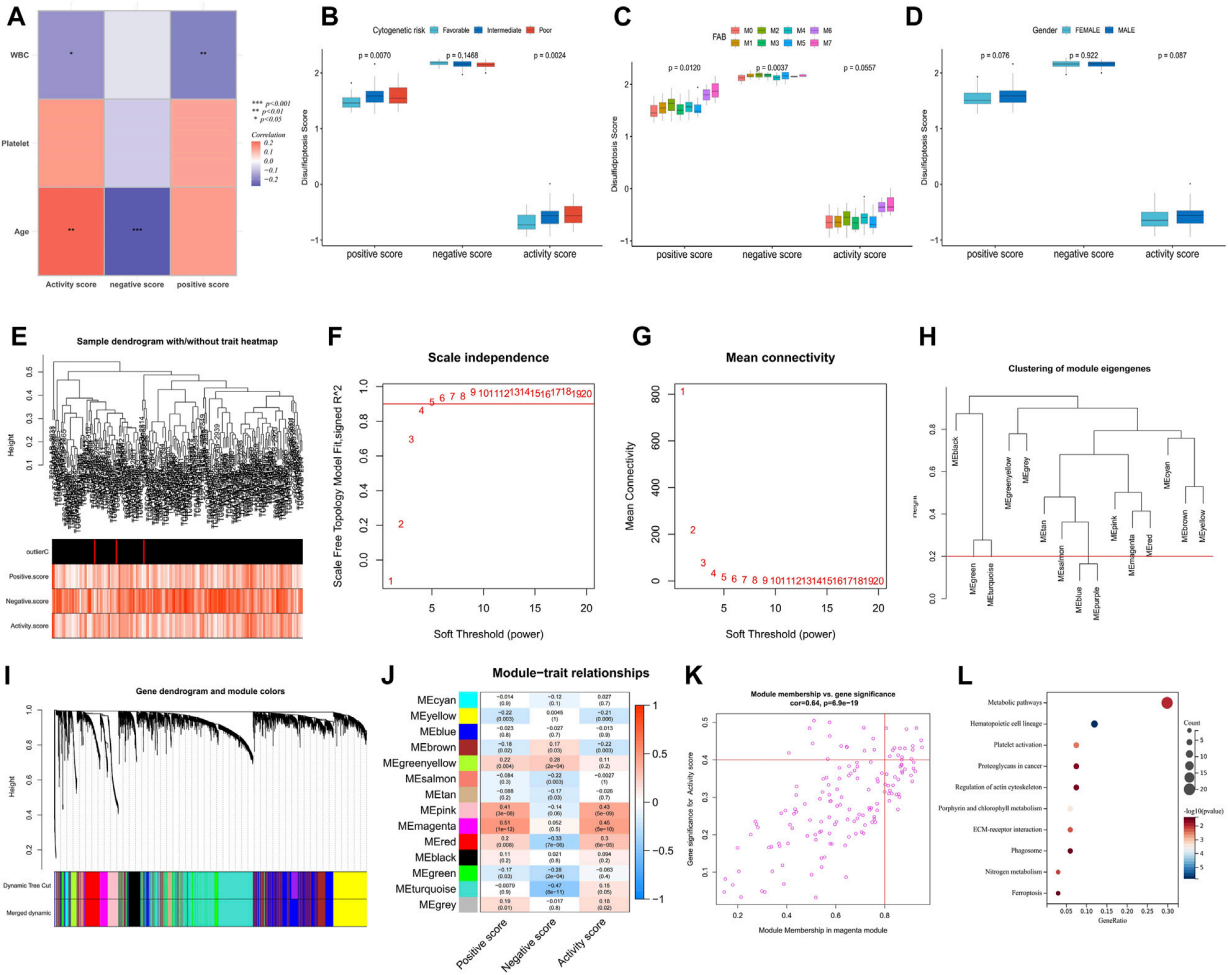
Analysis of clinical relevance of disulfidptosis scores and identification of potential DRGs. **(A)** Correlation analysis between disulfidptosis scores and clinicopathological factors. **(B–D)** Differences in disulfidptosis scores among clinicopathological factors. **(E)** Clustering dendrogram of AML samples. Color intensity was positively correlated with disulfidptosis scores. **(F,G)** Scale-free fit index **(F)** and average connectivity **(G)** analysis of various soft threshold powers. **(H)** the cluster of module feature genes. The red line indicates the cutting height (0.2). **(I)** Dendrogram of clustering based on different measures (1-TOM). **(J)** Heatmap of correlation between module signature genes and disulfidptosis score. Each cell contains a *p*-value and a correlation coefficient. **(K)** Scatter plot of module characteristic genes associated with disulfidptosis activity score in magenta modules. **(L)** KEGG enrichment analysis of magenta module genes. (*p < 0.05; **p < 0.01; ***p < 0.001).

Correlation analysis of immune infiltration showed that a high disulfidptosis activity score was associated with reduced infiltration of M2 macrophages and memory B cells, and increased infiltration of neutrophils. Conversely, high disulfidptosis negative scores were associated with increased infiltration of memory B cells, M1 macrophages, mast cells, and resting CD4^+^ T cells, along with reduced infiltration of monocytes and activated CD4^+^ T cells (Figure 2H). Analysis of TME characteristics demonstrated that higher disulfidptosis activity scores were associated with higher immune and stromal scores and lower tumor purity, whereas the opposite trend was observed for negative scores (Figure 2I). This suggests the involvement of disulfidptosis in antitumor responses within the AML TME. Moreover, the disulfidptosis activity score was significantly positively correlated with the expression of most immune checkpoints, with the opposite seen for the negative score (Figure 2J). Pathway analysis revealed that the activity score was positively correlated with the enrichment score of most tumor marker pathways (Figure 2K). Among these pathways, proliferation-related signaling pathways like *MYC* targets V1/V2 and DNA repair were negatively correlated with the activity score, indicating a potential inhibitory effect of disulfidptosis on cell viability. Correlation analysis with clinicopathological factors showed that white blood cell (WBC) count exhibited a negative correlation with both the positive score and activity score, while age showed a positive correlation with the activity score and a negative correlation with the negative score (Figure 3A). In the comparison of categorical variables, the activity score and positive score increased with an increase in cytogenetic risk, with the highest values observed in the French-American-British (FAB) classification of M6 and M7 (Figures 3B, C). Moreover, disulfidptosis scores did not show significant differences among patients (Figure 3D).

### Identification of potential DRGs and signaling pathways

The weighted correlation network analysis (WGCNA) analysis was performed on The Cancer Genome Atlas—Acute Myeloid Leukemia (TCGA-LAML) dataset to identify additional potential DRGs. The cluster dendrogram displayed an increase in color depth corresponding to the magnitude of the disulfidptosis score (Figure 3E). Figures 3F, G show the scale-free fit exponent and average connectivity analysis for various soft threshold powers. The blue and purple module feature genes were combined with a cut height of 0.2 (Figures 3H, I). A soft threshold power of *β* = 5 (unscaled *R2* = 0.9) was selected to categorize the top 5,000 genes, sorted by standard deviation, into 14 independent co-expression modules (Figure 3J). The correlation plot of the module-trait relationship indicated that the magenta gene module, comprising 152 genes, exhibited the highest correlation with the disulfidptosis activity score (Figures 3J, K; Supplementary Table S2). KEGG analysis highlighted that these genes were mainly enriched in signaling pathways such as metabolic pathways, hematopoietic cell lineage, platelet (PLT) activation, and proteoglycans in cancer (Figure 3L).

### Identification of disulfidptosis-related molecular subtypes and analysis of their differences in biological characteristics

**FIGURE 4:**
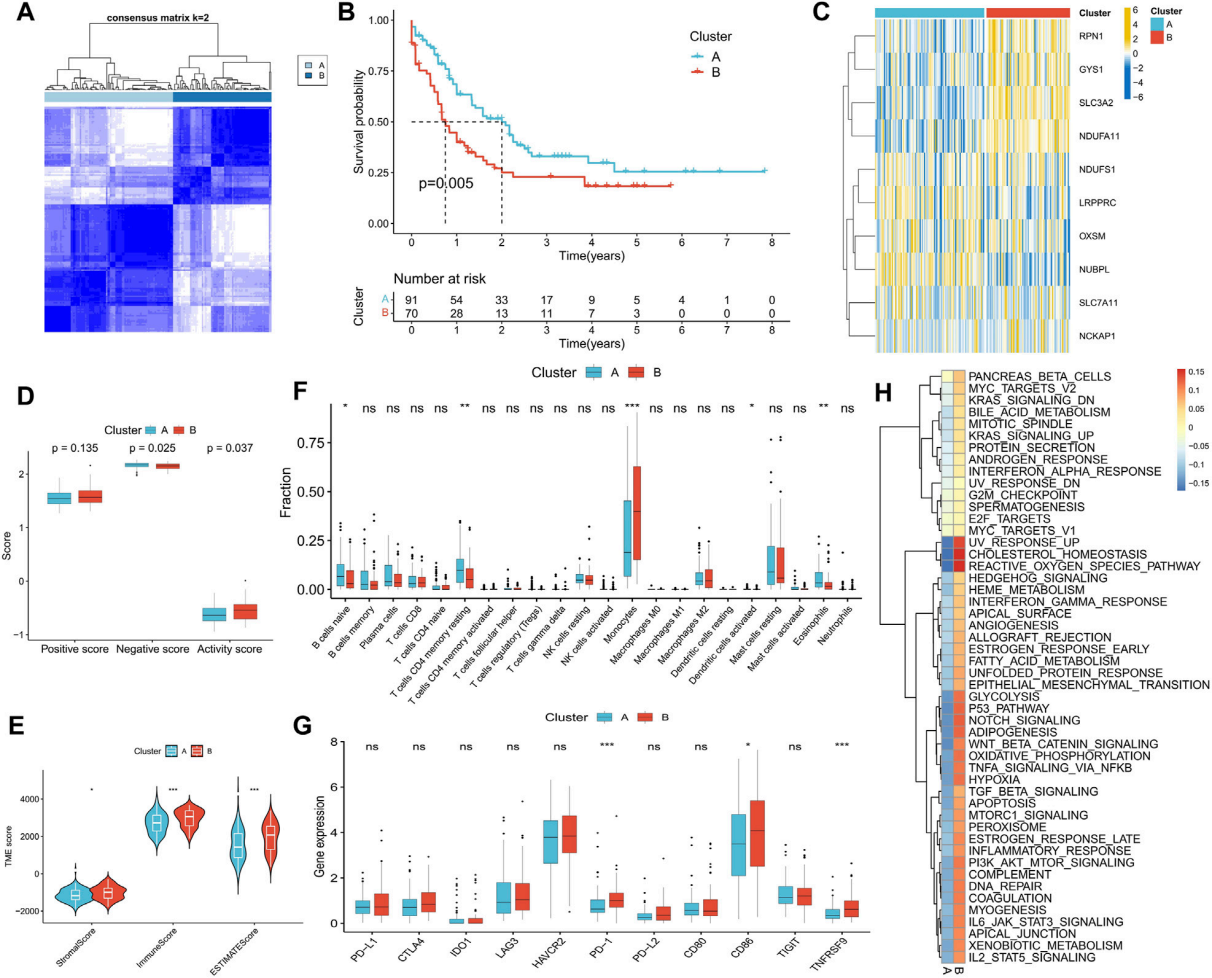
Identification of disulfidptosis-related molecular subtypes and analysis of differences in biological characteristics between subtypes. **(A)** Two molecular subtypes were identified by consensus clustering. **(B)** Survival analysis between subtypes. **(C)** Heatmap shows the expression characteristics of DRGs between subtypes. **(D–H)** Differences in disulfidptosis score **(D)**, TME score **(E)**, immune cell infiltration **(F)**, immune checkpoint expression **(G)**, and tumor marker gene set score **(H)** between subtypes. (*p < 0.05; **p < 0.01; ***p < 0.001).

We performed a consensus cluster analysis based on the expression of DRGs retrieved in the study of Gan et al. According to the distribution characteristics of the cluster plots and considering the small size of samples in the TCGA-LAML cohort (Supplementary Figure S2). We chose cluster number 2 as optimal. Two disulfidptosis-related molecular subtypes, Cluster A and Cluster B, were thus identified (Figure 4A). Cluster B demonstrated a significantly worse prognosis than Cluster A (Figure 4B). The expression of *RPN1*, *GYS1*, *SLC3A2*, and *NDUFA11* was significantly higher in Cluster B than in Cluster A (Figure 4C). Additionally, Cluster A exhibited a higher disulfidptosis negative score, while Cluster B displayed a higher activity score (Figure 4D). TME analysis indicated that Cluster B possessed higher stromal and immune scores (Figure 4E), primarily due to a greater proportion of monocyte infiltration. In contrast, Cluster A was enriched in more naive B cells, resting memory CD4^+^ T cells, and eosinophils (Figure 4F). Higher expression of *PD-1*, *TNFRSF9*, and *CD86* was observed in Cluster B, suggesting the potential presence of immunosuppression in this subtype (Figure 4G). Difference analysis results showed that the activity of 50 tumor marker pathways in Cluster B exceeded that in Cluster A (Figure 4H).

### Prognostic predictive value of DRG analysis

**FIGURE 5:**
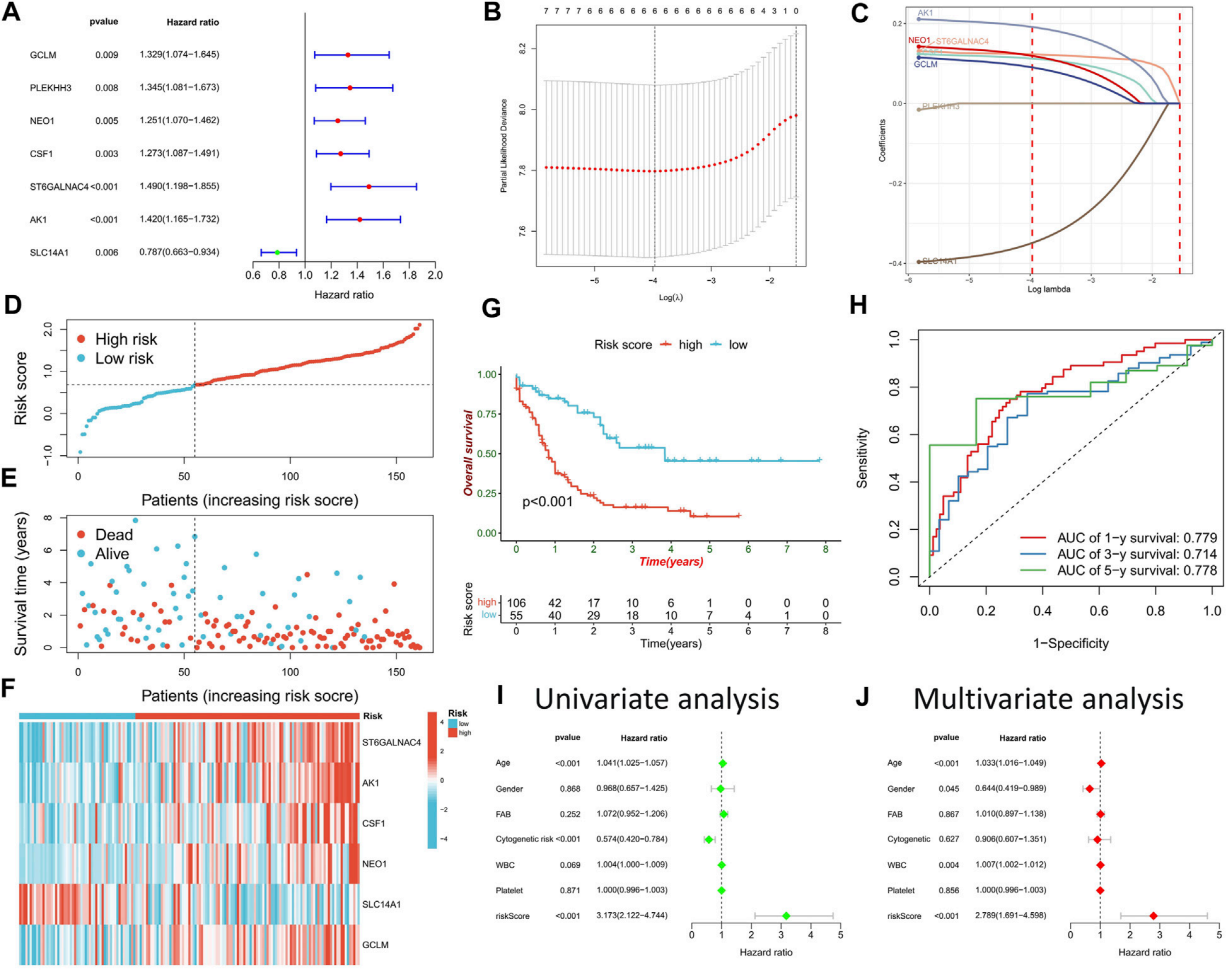
Construction of risk scoring model. **(A)** Cox regression analysis was used to identify DRGs significantly associated with prognosis. **(B)** The penalty coefficient of the minimum 10-fold cross-validation error point was calculated to determine the corresponding model gene. **(C)** Determination of model gene coefficients. **(D–F)** Based on the optimal cut-off value, patients in the TCGA-LAML cohort were divided into high- and low-risk score groups **(D)**, the survival status distribution **(E)**, and model gene expression **(F)** in high- and low-risk score groups. **(G)** Survival analysis between high- and low-risk score groups. **(H)** Time-dependent ROC curve analysis of risk scores. **(I,J)** Univariate and multivariate Cox regression analysis of clinicopathological factors and risk score.

Univariate Cox regression analysis on potential DRGs identified by WGCNA analysis revealed significant correlations between AML prognosis and *GCLM*, *PLEKHH3*, *NEO1*, *CSF1*, *ST6GALNAC4*, *AK1*, and *SLC14A1* (Figure 5A). To reduce dimensionality and construct a prognostic risk score model, LASSO regression analysis was performed using six genes, excluding *PLEKHH3* (Figures 5B, C) (Supplementary Table S3). Patients with AML were stratified into high- and low-risk score groups based on the optimal cut-off value (Figure 5D). Compared to the low-risk score group, the high-risk score group exhibited a higher number of deceased patients (Figure 5E), higher expressions of *GCLM*, *NEO1*, *CSF1*, *ST6GALNAC4*, and *AK1*, and lower expression of *SLC14A1* (Figure 5F). Survival analysis demonstrated that the high-risk score group had a significantly worse prognosis than the low-risk score group (Figure 5G). ROC curve analysis revealed high area under the curve (AUC) values for the risk score at 1, 3, and 5 years (0.779, 0.714, and 0.778, respectively), indicating the robust prognostic value of the risk score (Figure 5H). Univariate and multivariate Cox regression analyses confirmed the risk score as an independent factor for predicting AML prognosis (*p* < 0.001) (Figures 5I, J).

### Validation of prognostic ability of risk score model and clinical nomogram construction

**FIGURE 6:**
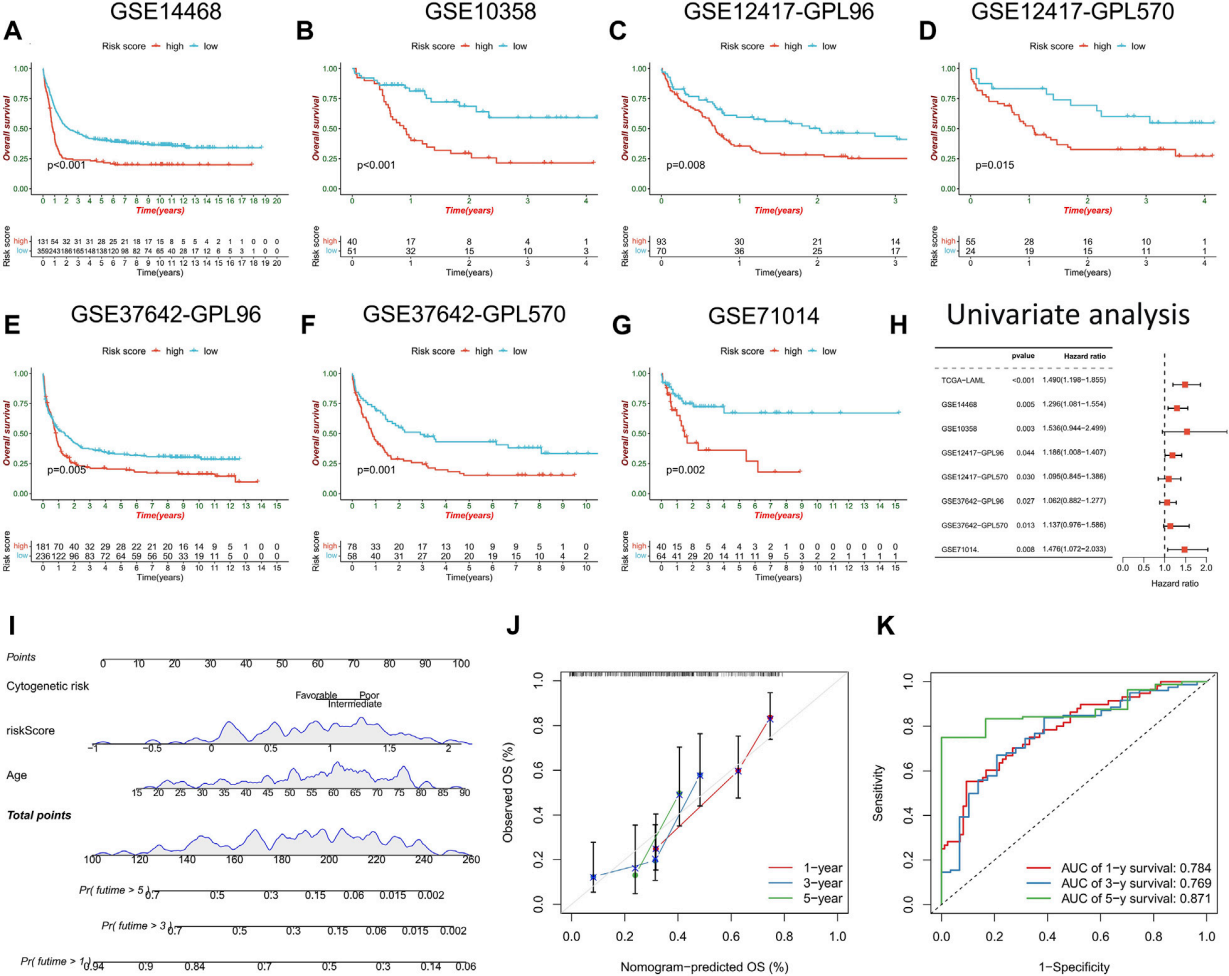
Validation of risk score model and construction of nomogram. **(A–G)** survival analysis between high- and low-risk score groups in the validation cohorts. **(H)** Univariate regression analysis were performed to evaluate the prognostic predictive power of risk score model in the training cohort and the validation cohorts. **(I)** Nomogram constructed by risk score combined with clinicopathological factors to predict OS of AML patients. **(J)** time-dependent calibration curve to verify the predictive power of the nomogram. **(K)** ROC curve analysis of nomogram.

The prognostic prediction ability of the risk score model was further validated across seven AML cohorts, with the high-risk score group consistently exhibiting a significantly worse prognosis than the low-risk score group (Figures 6A–G). Univariate Cox regression analysis confirmed the prognostic power of risk score model (*p* < 0.05) (Figure 6H). A clinical nomogram was constructed by combining clinicopathological factors significantly associated with AML prognosis, namely, age and cytogenetic risk (Figure 6I). Calibration curve analysis demonstrated the consistency between observed overall survival (OS) and predicted OS (Figure 6J). ROC curve analysis showed high AUC values for the nomogram at 1, 3, and 5 years (0.784, 0.769, and 0.871, respectively), confirming its strong prognostic value (Figure 6K).

### Immunological features, pathway activity, genomic traits, and clinicopathological factors differences between high- and low-risk score groups

**FIGURE 7:**
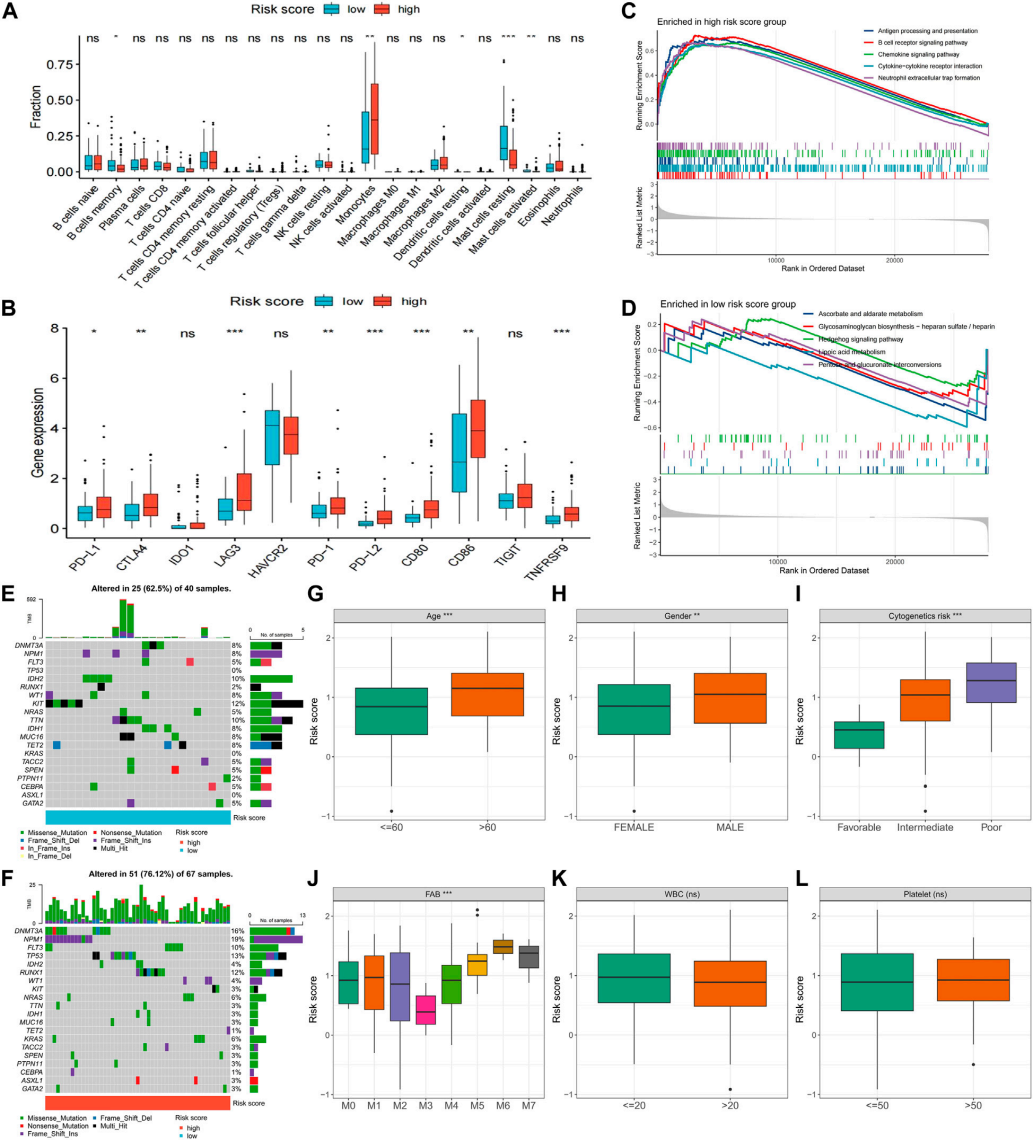
Differences in clinical characteristics and biological factors between high- and low-risk score groups. Differences in immune cell infiltration **(A)**, immune checkpoint expression **(B)**, enriched pathways **(C,D)**, and somatic mutation frequency **(E,F)** between high- and low-risk score groups. Differences in risk scores between subgroups with different clinicopathological factors **(G–L)**. FAB, French–American–British; WBC, white blood cell. (*p < 0.05; **p < 0.01; ***p < 0.001).

Further exploration was conducted to explore potential reasons for the significant prognostic differences between the high- and low-risk score groups. The high-risk score group had a higher count of monocytes, while the low-risk score group had more memory B cells and resting and activated mast cells (Figure 7A). Notably, immune checkpoint expression, including *PD-L1*, *CTLA1*, *LAG3*, *PD-1*, *PD-L2*, *CD80*, *CD86*, and *TNFRSF9*, was significantly upregulated in the high-risk score group (Figure 7B). GSEA enrichment analysis revealed heightened activity in immune-related signaling pathways, such as antigen processing and presentation, B cell receptor signaling, chemokine signaling, cytokine-cytokine receptor interaction, and neutrophil extracellular trap formation in the high-risk score group (Figure 7C). Conversely, metabolism-related pathways, including ascorbate and aldarate metabolism, glycosaminoglycan biosynthesis-heparan sulfate/heparin, hedgehog signaling, lipoic acid metabolism, and pentose and glucuronate interposition, were enriched in the low-risk score group (Figure 7D). The high-risk score group displayed a higher gene mutation rate, with *DNMT3A*, *NPM1*, *FLT3*, *TP53*, and *RUNX1* being the most frequently mutated genes (Figures 7E, F). Risk scores also significantly differed across various clinical characteristics, with patients of advanced age, male gender, and worse cytogenetic risk demonstrating higher risk scores (Figures 7G–I). In terms of the French-American-British (FAB) classification, patients with the M3 type had the lowest risk scores, while patients with the M5-M7 type exhibited higher risk scores (Figure 7J). Notably, risk scores did not differ significantly across white blood cells (WBC) and platelet (PLT) count groups (Figures 7K, L).

### Sensitivity of chemotherapy and immunotherapy differences between high- and low-risk score groups

**FIGURE 8:**
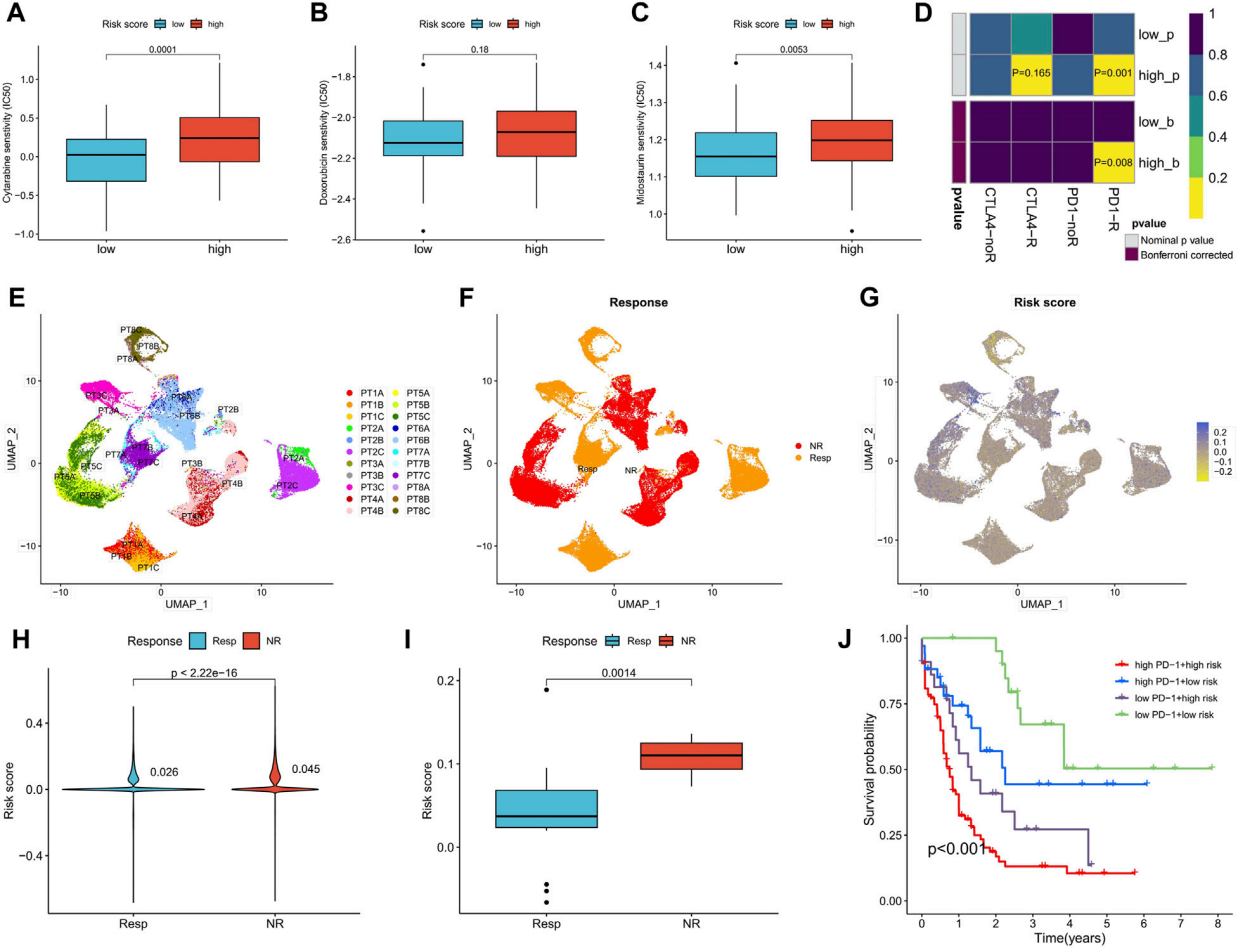
Differences in chemotherapy sensitivity and immunotherapy response between high- and low-risk score groups. **(A–C)** Sensitivity prediction of cytarabine, doxorubicin, and midostaurin for AML in high- and low-risk score groups. **(D)** Prediction of response to anti-PD-1 and anti-CTAL4 immunotherapy in different risk score groups. **(E)** UMAP analysis of the AML single-cell sequencing dataset GSE198052 shows the distribution of AML cell expression (indicated by different colors) in different patients. **(F)** Distribution of AML cells in patients with and without response to anti-PD-1 therapy. **(G)** Risk scores for all AML cells of patients with and without response to anti-PD-1 therapy. **(H,I)** Analysis of differences in all AML cellular risk scores **(H)** and mean risk scores **(I)** between patients who responded and those who did not respond to anti-PD-1 therapy. **(J)** Survival analysis of TCGA-LAML patients grouped according to risk score and PD-1 expression. PT, The patient.

Sensitivity to commonly used AML drugs was predicted, with the low-risk score group exhibiting lower IC50 values for cytarabine and midostaurin, indicating greater sensitivity to these drugs. No significant difference in sensitivity to doxorubicin was observed (Figures 8A–C). Predictive results for immunotherapy showed that the high-risk score group was more responsive to anti-PD-1 treatment, with significantly upregulated *PD-1* expression (Figure 8D). We further analyzed the differences in risk scores among different patients in a set of AML single-cell sequencing datasets after anti-PD-1 treatment. Figures 8E–G shows AML cell distribution, treatment response versus non-response population, and expression levels of risk scores in patients with AML, respectively. Analysis of the risk scores revealed that the single-cell risk score and average risk score were higher in patients with AML who did not respond to anti-PD-1 therapy compared to those who responded (Figures 8H, I). In the TCGA-LAML cohort, patients with both high *PD-1* expression and high-risk scores exhibited the worst prognosis, while those with low levels of both had the best prognosis (Figure 8J). These results suggest that the risk score can predict the sensitivity of patients to chemotherapy and immunotherapy.

### Validation in a real-world clinical cohort

**FIGURE 9:**
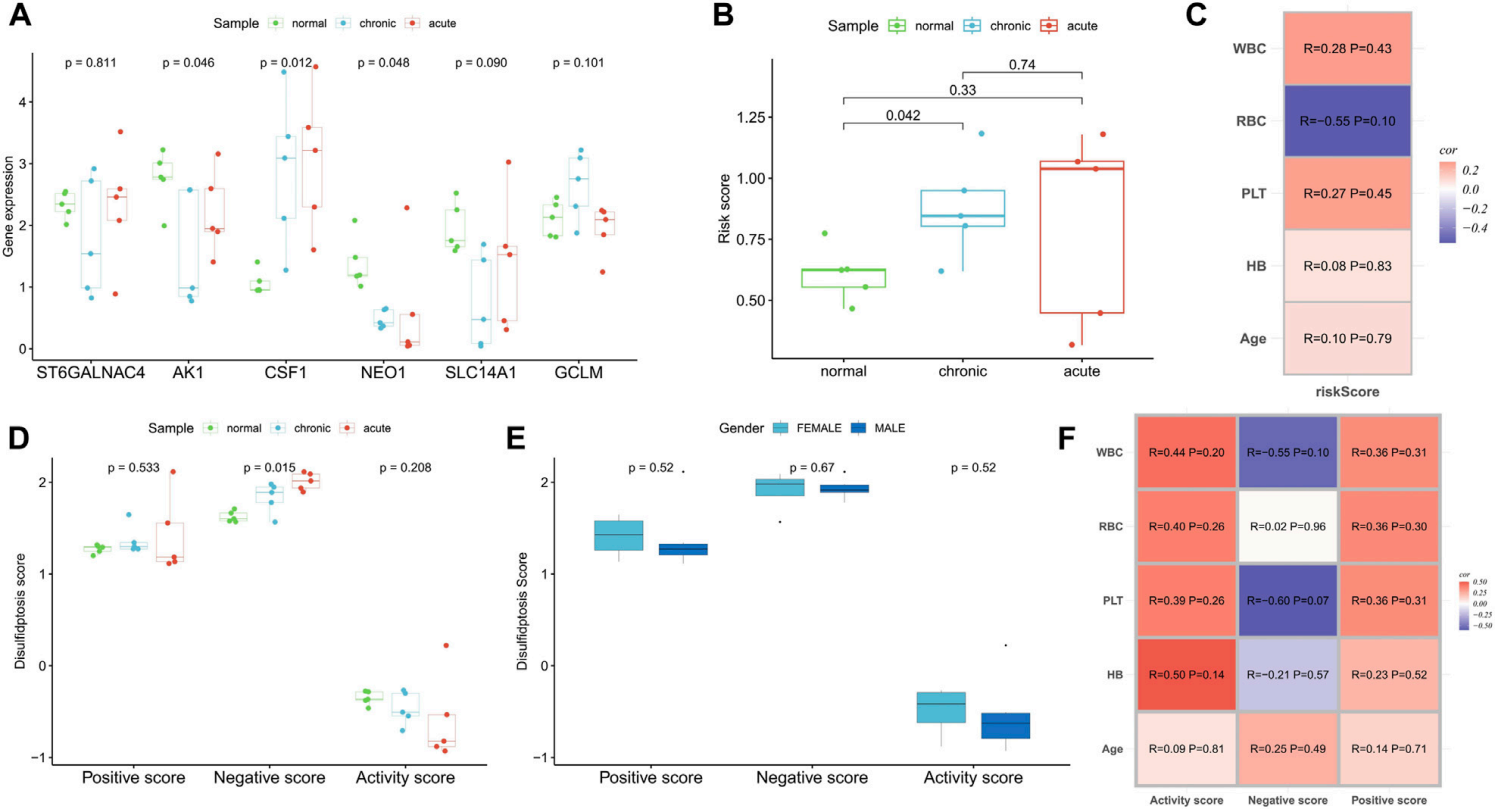
Clinical cohort was used to verify the correlation of disulfidptosis scores and risk score with disease progression. **(A,B)** Differences in the expression of risk scoring model genes **(A)** and risk score **(B)** among normal samples, myeloid leukemia chronic-phase samples and myeloid leukemia acute-phase samples. **(C)** Correlation analysis between risk score and clinicopathological factors. **(D)** Differences in disulfidptosis scores among normal samples, myeloid leukemia chronic-phase samples and myeloid leukemia acute-phase samples. **(E)** Differences in disulfidptosis scores between patients with different genders. **(F)** Correlation analysis between disulfidptosis scores and clinicopathological factors.

In a real-world clinical cohort, transcriptome sequencing was performed on samples from five normal individuals, five myeloid leukemia chronic-phase patients, and five myeloid leukemia acute-phase patients. Compared to the normal samples, the expression of *CSF1* was upregulated in the chronic and acute phase samples, while *AK1*, *NEO1*, and *SLC14A1* were downregulated. The expression of *GCLM* was upregulated in the chronic phase samples, while *ST6GALNAC4* showed no significant change (Figure 9A). Risk scores increased with the progression of myeloid leukemia (Figure 9B). Correlation analysis with clinicopathological factors indicated positive correlations between the risk score and WBC, PLT, hemoglobin (HB), and age, while red blood cell (RBC) count showed a negative correlation (Figure 9C). Although the small sample size did not yield significant correlations (*p* < 0.05), disulfidptosis negative scores increased and activity scores decreased with the progression of myeloid leukemia (Figure 9D), confirming the presence of disulfidptosis resistance. Notably, there were no significant differences in disulfidptosis scores between gender groups (Figure 9E). Positive and active scores were positively correlated with WBC, RBC, PLT, HB, and age, while negative scores exhibited negative correlations with WBC, PLT, and HB (Figure 9F).

## Discussion

Evasion of cell death plays a crucial role in the occurrence and development of tumors, contributing significantly to drug resistance (Strasser and Vaux, 2020). The rapid proliferation of AML cells seriously affects the hematopoietic and immune systems of patients, leading to complications like bleeding and infections that pose substantial health risk (Shimony et al., 2023). Tumor cells benefit from heightened energy metabolism and rely on antioxidants such as glutathione to scavenge reactive oxygen species generated during metabolism, protecting the cells from oxidative damage (Hole et al., 2011). Disulfidptosis, a novel metabolism-related cell death mechanism proposed by Liu et al. (2023), relies on glutamate intake through xCT and is influenced by glucose scarcity. Our study has revealed abnormal expression patterns of DRGs in AML, showing a strong correlation with patient prognosis. It has also identified two subtypes related to disulfidptosis with significant differences in biological characteristics. Our DRG-based risk score model can accurately predict the prognosis and treatment sensitivity of patients with AML.

All 10 DRGs were significantly upregulated in AML samples, with all except *NUBPL* serving as prognostic risk factors, indicating the potential role of DRGs in carcinogenesis. Patients with higher disulfidptosis positive and activity scores exhibited a poorer prognosis, while the opposite was true for negative scores. AML cells with heightened activity scores may be more sensitive to disulfidptosis. Analysis of single-cell data also confirmed the overexpression of disulfidptosis suppressor genes in AML malignant cells and revealed a higher ferroptosis inhibition score. These findings align with our clinical cohort analysis, suggesting that AML progression is accompanied by greater resistance to disulfidptosis. Thus, patients with a poor prognosis might benefit from disulfidptosis induction to inhibit AML cell activity. Moreover, a significant negative correlation between the TCA cycle, a vital glucose metabolism pathway, and activity scores suggests its pivotal role in inhibiting disulfidptosis in AML cells (Kreitz et al., 2019). A high activity score was associated with greater immune scores, higher immune checkpoint expression, and more active tumor marker pathways. These factors may contribute to the poorer prognosis seen in these patients.

Furthermore, based on the expression clustering of DRGs, we identified two molecular subtypes, with Cluster B patients exhibiting significantly worse prognosis. This group displayed higher expressions of *PD-1*, *TNFRSF9*, and *CD86*, along with increased enrichment scores for tumor marker pathways, distinguishing them from Cluster A patients.

Our analysis of potential DRGs, identified through WGCNA, revealed close associations with metabolic pathways. This highlights the intricate relationship between tumor metabolism and disulfidptosis. The DRG-based risk score model can accurately predict the prognosis of patients with AML and patients with high-risk scores exhibiting worse clinical outcomes. The risk score demonstrated robust predictive accuracy, with AUC values for 1-, 3-, and 5-year prognosis prediction exceeding 0.7. The model’s prognostic value was consistently confirmed across seven AML cohorts. Both univariate and multivariate regression analyses supported the risk score’s independence as a prognostic factor for AML. Moreover, our clinical cohort analysis confirmed a positive correlation between the risk score and myeloid leukemia progression. The nomogram, constructed by combining clinicopathological factors, offers an intuitive prediction of patient OS with high accuracy.

Next, we focused on the clinical and biological differences between high- and low-risk score groups. High-risk score patients exhibited more active immune-related signaling pathways, but their elevated immune checkpoint expression, particularly of *PD-1*, likely contributed to the poorer prognosis. These patients also showed a higher somatic mutation rate, a common characteristic of AML, with unfavorable cytogenetic risk, older age, and male gender associated with higher risk scores. The analysis indicated that patients with high-risk scores may respond well to anti-PD-1 therapy. Furthermore, their sensitivity to common chemotherapy agents such as cytarabine and midostaurin was lower. This underscores the importance of personalized treatment for patients with AML based on their risk scores.

In conclusion, our findings demonstrate a connection between DRGs and the occurrence and progression of AML. This connection is closely related to TME characteristics, immune status, and pathway activity. The DRG-based risk score model is a powerful tool for predicting prognosis, revealing differences in immune characteristics, and guiding personalized AML treatment. While this study has shed light on the expression patterns, potential biological mechanisms, and prognostic value of DRGs and pathways, further *in vivo* and *in vitro* experiments are necessary to elucidate their role in AML cells. Additionally, larger real-world cohorts will be required to validate the prognostic potential of the risk score model. Our future studies aim to provide deeper insights into the mechanisms of disulfidptosis in AML.

## Data Availability

The datasets presented in this study can be found in online repositories. The names of the repository/repositories and accession number(s) can be found in the article/Supplementary Material.

## References

[B1] Bhansali R. S. Pratz K. W. Lai C. (2023). Recent advances in targeted therapies in acute myeloid leukemia. J. Hematol. Oncol. 16, 29. 10.1186/s13045-023-01424-6 36966300 PMC10039574

[B2] Chen W. L. Wang J. H. Zhao A. H. Xu X. Wang Y. H. Chen T. L. (2014). A distinct glucose metabolism signature of acute myeloid leukemia with prognostic value. Blood 124, 1645–1654. 10.1182/blood-2014-02-554204 25006128 PMC5726328

[B3] Dixon S. Lemberg K. M. Lamprecht M. R. Skouta R. Zaitsev E. M. Gleason C. E. (2012). Ferroptosis: an iron-dependent form of nonapoptotic cell death. Cell 149, 1060–1072. 10.1016/j.cell.2012.03.042 22632970 PMC3367386

[B4] Galluzzi L. Vitale I. Aaronson S. A. Abrams J. M. Adam D. Agostinis P. (2018). Molecular mechanisms of cell death: recommendations of the nomenclature committee on cell death 2018. Cell Death Differ. 25, 486–541. 10.1038/s41418-017-0012-4 29362479 PMC5864239

[B5] Geeleher P. Cox N. Huang R. (2014). pRRophetic: an R package for prediction of clinical chemotherapeutic response from tumor gene expression levels. PloS one 9, e107468. 10.1371/journal.pone.0107468 25229481 PMC4167990

[B6] Goji T. Takahara K. Negishi M. Katoh H. (2017). Cystine uptake through the cystine/glutamate antiporter xCT triggers glioblastoma cell death under glucose deprivation. J. Biol. Chem. 292, 19721–19732. 10.1074/jbc.M117.814392 29038291 PMC5712613

[B7] Hole P. S. Darley R. L. Tonks A. (2011). Do reactive oxygen species play a role in myeloid leukemias? Blood 117, 5816–5826. 10.1182/blood-2011-01-326025 21398578

[B8] Jiang A. Wang J. Liu N. Zheng X. Li Y. Ma Y. (2022). Integration of single-cell RNA sequencing and bulk RNA sequencing data to establish and validate a prognostic model for patients with lung adenocarcinoma. Front. Genet. 13, 833797. 10.3389/fgene.2022.833797 35154287 PMC8829512

[B9] Koppula P. Zhang Y. Shi J. Li W. Gan B. (2017). The glutamate/cystine antiporter SLC7A11/xCT enhances cancer cell dependency on glucose by exporting glutamate. J. Biol. Chem. 292, 14240–14249. 10.1074/jbc.M117.798405 28630042 PMC5572906

[B10] Koppula P. Zhuang L. Gan B. (2021). Cystine transporter SLC7A11/xCT in cancer: ferroptosis, nutrient dependency, and cancer therapy. Protein and Cell 12, 599–620. 10.1007/s13238-020-00789-5 33000412 PMC8310547

[B11] Kreitz J. Schönfeld C. Seibert M. Stolp V. Alshamleh I. Oellerich T. (2019). Metabolic plasticity of acute myeloid leukemia. Cells 8, 805. 10.3390/cells8080805 31370337 PMC6721808

[B12] Langfelder P. Horvath S. (2008). WGCNA: an R package for weighted correlation network analysis. BMC Bioinforma. 9, 559. 10.1186/1471-2105-9-559 PMC2631488 19114008

[B13] Li S. Q. Liu J. Zhang J. Wang X. L. Chen D. Wang Y. (2020). Transcriptome profiling reveals the high incidence of hnRNPA1 exon 8 inclusion in chronic myeloid leukemia. J. Adv. Res. 24, 301–310. 10.1016/j.jare.2020.04.016 32405436 PMC7210475

[B14] Liu X. Nie L. Zhang Y. Yan Y. Wang C. Colic M. (2023). Actin cytoskeleton vulnerability to disulfide stress mediates disulfidptosis. Nat. Cell Biol. 25, 404–414. 10.1038/s41556-023-01091-2 36747082 PMC10027392

[B15] Liu X. Olszewski K. Zhang Y. Lim E. W. Shi J. Zhang X. (2020). Cystine transporter regulation of pentose phosphate pathway dependency and disulfide stress exposes a targetable metabolic vulnerability in cancer. Nat. Cell Biol. 22, 476–486. 10.1038/s41556-020-0496-x 32231310 PMC7194135

[B16] Mohammad R. M. Muqbil I. Lowe L. Yedjou C. Hsu H. Y. Lin L. T. (2015). Broad targeting of resistance to apoptosis in cancer. Semin. Cancer Biol. 35, S78-S103–s103. 10.1016/j.semcancer.2015.03.001 25936818 PMC4720504

[B17] Newman A. Liu C. L. Green M. R. Gentles A. J. Feng W. Xu Y. (2015). Robust enumeration of cell subsets from tissue expression profiles. Nat. methods 12, 453–457. 10.1038/nmeth.3337 25822800 PMC4739640

[B18] Pardieu B. Pasanisi J. Ling F. Dal Bello R. Penneroux J. Su A. (2022). Cystine uptake inhibition potentiates front-line therapies in acute myeloid leukemia. Leukemia 36, 1585–1595. 10.1038/s41375-022-01573-6 35474100 PMC12860451

[B19] Shimony S. Stahl M. Stone R. M. (2023). Acute myeloid leukemia: 2023 update on diagnosis, risk-stratification, and management. Am. J. Hematol. 98, 502–526. 10.1002/ajh.26822 36594187

[B20] Shin C. S. Mishra P. Watrous J. D. Carelli V. D'Aurelio M. Jain M. (2017). The glutamate/cystine xCT antiporter antagonizes glutamine metabolism and reduces nutrient flexibility. Nat. Commun. 8, 15074. 10.1038/ncomms15074 28429737 PMC5413954

[B21] Strasser A. Vaux D. L. (2020). Cell death in the origin and treatment of cancer. Mol. Cell 78, 1045–1054. 10.1016/j.molcel.2020.05.014 32516599

[B22] Subramanian A. Tamayo P. Mootha V. K. Mukherjee S. Ebert B. L. Gillette M. A. (2005). Gene set enrichment analysis: a knowledge-based approach for interpreting genome-wide expression profiles. Proc. Natl. Acad. Sci. 102, 15545–15550. 10.1073/pnas.0506580102 16199517 PMC1239896

[B23] van Galen P. Hovestadt V. Wadsworth Ii M. H. Hughes T. K. Griffin G. K. Battaglia S. (2019). Single-cell RNA-seq reveals AML hierarchies relevant to disease progression and immunity. Cell 176, 1265–1281. 10.1016/j.cell.2019.01.031 30827681 PMC6515904

[B24] Wilkerson M. Hayes D. (2010). ConsensusClusterPlus: a class discovery tool with confidence assessments and item tracking. Bioinforma. Oxf. Engl. 26, 1572–1573. 10.1093/bioinformatics/btq170 PMC2881355 20427518

[B25] Yoshihara K. Shahmoradgoli M. Martínez E. Vegesna R. Kim H. Torres-Garcia W. (2013). Inferring tumour purity and stromal and immune cell admixture from expression data. Nat. Commun. 4, 2612. 10.1038/ncomms3612 24113773 PMC3826632

[B26] Zhang C. Liu X. Jin S. Chen Y. Guo R. (2022). Ferroptosis in cancer therapy: a novel approach to reversing drug resistance. Mol. Cancer 21, 47. 10.1186/s12943-022-01530-y 35151318 PMC8840702

[B27] Zheng H. Li Y. Zhao Y. Jiang A. (2023). Single-cell and bulk RNA sequencing identifies T cell marker genes score to predict the prognosis of pancreatic ductal adenocarcinoma. Sci. Rep. 13, 3684. 10.1038/s41598-023-30972-7 36878969 PMC9988929

