# Supplementary material for: Development and validation of a disulfidptosis-related scoring system to predict clinical outcome and immunotherapy response in acute myeloid leukemia by integrated analysis of single-cell and bulk RNA-sequencing
Source: Front Pharmacol. 2023 Nov 20;14:1272701. doi: 10.3389/fphar.2023.1272701 (PMC10694296; doi:10.3389/fphar.2023.1272701)

**Figure S1. Prognostic analysis of disulfidptosis-related genes (DRGs).**


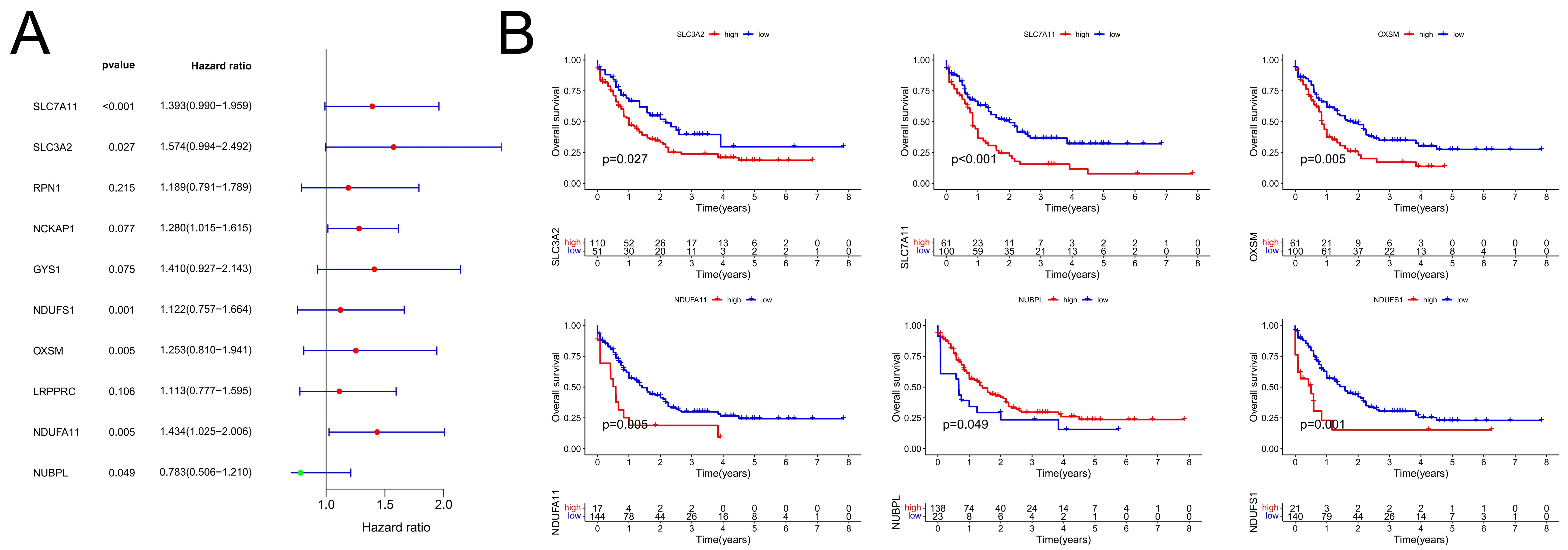


(A) Univariate Cox regression analysis of DRGs expression. (B) K-M curve analysis of DRGs expression;

**Figure S2. Cluster plots with cluster numbers of 2-9 in the consensus cluster analysis.**


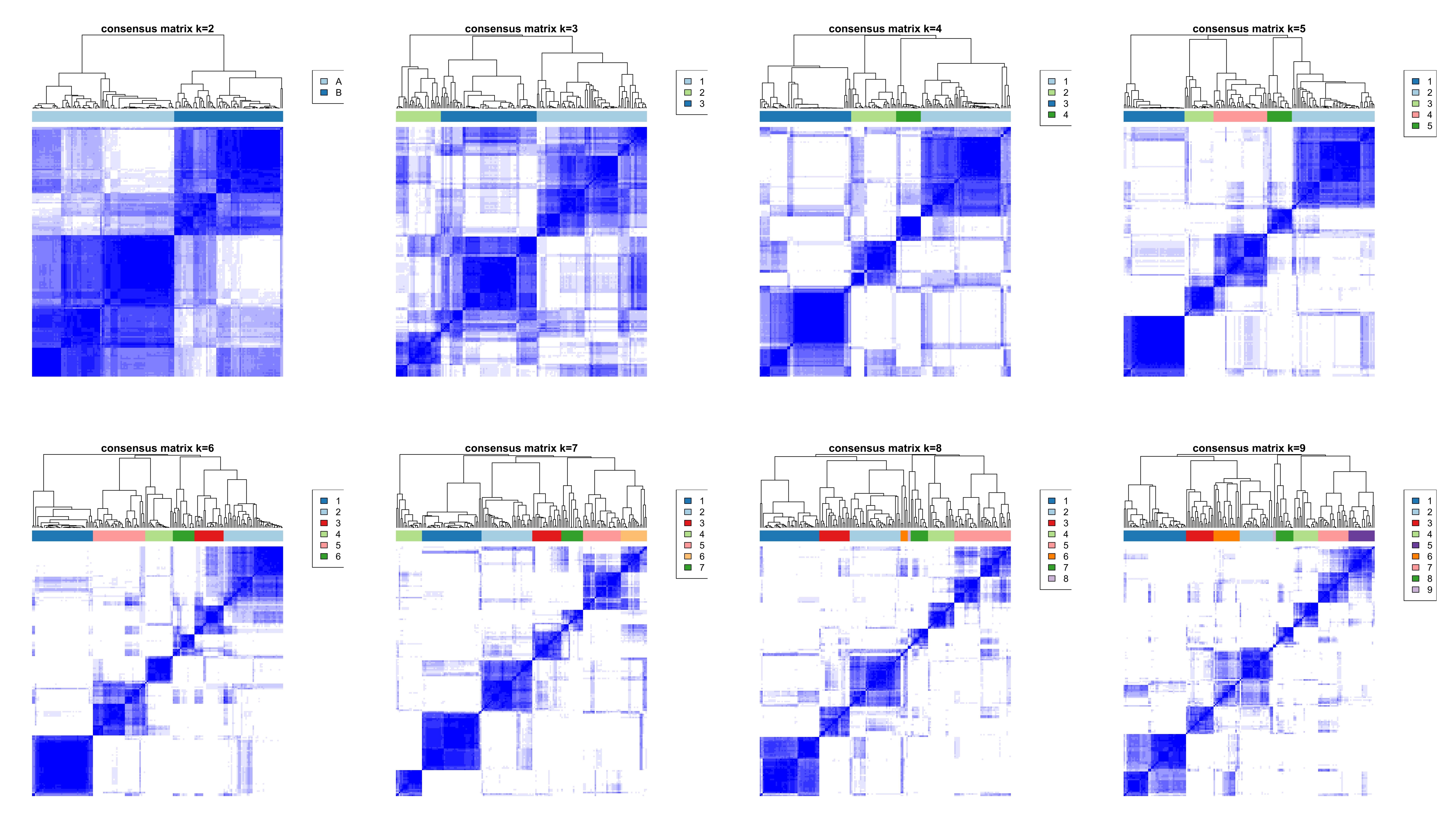

Supplement: Supplementary file 1 [file DataSheet2.docx]
